# Suicide: An Un-resolved and Under-recognized Health Problem among People of Ilam Province (The Most Popular Suicide Spot in Iran)

**Published:** 2017-09

**Authors:** Aziz KASSANI, Abdolvahab BAGHBANIAN, Walieh MENATI

**Affiliations:** 1. Dept. of Community Medicine, School of Medicine, Dezful University of Medical Sciences, Dezful, Iran; 2. Health Systems and Global Populations, Faculty of Health Sciences, University of Sydney, Sydney, Australia; 3. Prevention of Psychosocial Injuries Research Center, Ilam University of Medical Sciences, Ilam, Iran

## Dear Editor-in-Chief

Suicide is a serious, yet preventable, health concern that effects and affects individuals, families, and communities all around the world. It is considered to be one of the leading causes of mortality in western countries with evidence showing that it is the second underlying cause of death among individuals aged 15 to 19 yr old in different communities (1, 2). The global rate of suicides is estimated at 16 per 100000 or one suicide every 40 sec, representing more than 800000 committed suicides per year (excluding attempted suicides or non-fatal suicidal behavior) (3). The global suicide rate has increased by 60% over the last 45 yr, with population aged 15–34 yr being the most vulnerable to being the victims of suicide (3). According WHO, an estimated 1.53 million individuals will die from suicide in 2020 (2, 3). Such a tragic crisis is, however, under-recognized, under-reported and under-treated across communities around the world.

The average suicide rate in Eastern Mediterranean Region including Iran (5.6 per 100000 for suicide) is lower than that of the developed countries (4). However, the trend of suicide rate has recently increased in Iran (5). The residents of Ilam Province, located in the west of Iran, are estimated to have the highest incidence rate of suicides in the country (19.53 cases/100000 population) (6), almost four times higher than that of the country’s average as of 2012 (5.2 per 100000 population) (5). The Ilam Province is now regarded as a high-risk area for suicide relative to other areas in the country (5, 6) and this trend is expected to continue to rise in future.

Through a study conducted between 2010 and 2013 in Ilam Province, data from the systematic registration suicide data system (SRSD) provided by Ilam University of Medical Sciences and data from the Ilam Organization of Forensic/Legal Medicine were used to investigate the suicide incidence rate in the region. A sample of 628 cases was reviewed. The mean age of the suicide victims was 27.83±11.23 yr, with a minimum and maximum of 10 and 78 yr old, respectively. The majority of victims were female (n=498, 79.30%) and, almost one-third of them (n=197, 31.36%) were illiterate or had primary education only. The age-standardized incidence rate (ASIR) of reported suicides was 20.48 per 100000 (CI95%: 19.75–21.10), 21.22 per 100000 (CI95%: 20.13–22.48), 21.10 per 100000 (CI95%: 20.01–22.44) and 22.23 per 100000 (CI95%: 21.31–23.19) during the period of 2010 to 2013, respectively. Females were found to have the highest ASRI of suicide with 29.09 cases per 100000 women (CI95%: 25.47–32.16) in 2013. In addition, female ASRI of suicide was much higher than male during the period of study (*P*=0.01).The highest unstandardized incidence rate (IR) was attributed to the city of Ilam (29.42 per 100000), city of Aivan (27.56 per 100000), city of Shirvan (24.22 per 100000) and city of Malekshahi (22.23 per 100000). There was also an increased trend in the incidence rate of suicide in the Ilam Province during the study period. The suicide incidence rate in Ilam Province was relatively higher than other provinces in the country (5, 6), reflecting the magnitude of this public health issue and the serious problems it continues to cause.

National leadership and shared vision are required to initiate a shift in individuals and communities’ thinking, behavior and decision about health issues including the suicide prevention: Suicide prevention should be viewed through a network of individuals and stakeholders who work collaboratively to develop and achieve shared goals (1). While health sector may have responsibility for a suicide prevention intervention, an effective one may lie in the domain of sectors. It should be adequately funded and supported by the public and private sectors that can address and prioritize education, awareness, treatment and community engagement. The prevention should also include strategies aimed at protecting high-risk groups including encouraging people with mental illness to seek psychological/rehabilitation advice (1, 7).

In particular, health policy-makers and care professionals are recommended to pay more attention to suicide and it’s risk factors while planning for the population health in Ilam Province as it is a highly prevalent area for suicide in Iran. Future longitudinal and cohort studies may look into the possible risk factors of suicide in this hotspot area of suicide.
